# Methionine sulfoxides in serum proteins as potential clinical biomarkers of oxidative stress

**DOI:** 10.1038/srep38299

**Published:** 2016-12-08

**Authors:** Satoko Suzuki, Yoshio Kodera, Tatsuya Saito, Kazumi Fujimoto, Akari Momozono, Akinori Hayashi, Yuji Kamata, Masayoshi Shichiri

**Affiliations:** 1 Department of Endocrinology, Diabetes and Metabolism, Kitasato University School of Medicine, 1-15-1 Kitasato, Minami-ku, Sagamihara, Kanagawa 252-0374, Japan; 2 Laboratory of Biophysics, Department of Physics, Kitasato University School of Science, 1-15-1 Kitasato, Minami-ku, Sagamihara, Kanagawa 252-0373, Japan; 3 Center for Disease Proteomics, Kitasato University School of Science, 1-15-1 Kitasato, Minami-ku, Sagamihara, Kanagawa 252-0373, Japan

## Abstract

Oxidative stress contributes to the pathophysiology of a variety of diseases, and circulating biomarkers of its severity remains a topic of great interest for researchers. Our peptidomic strategy enables accurate and reproducible analysis of circulating proteins/peptides with or without post-translational modifications. Conventional wisdom holds that hydrophobic methionines exposed to an aqueous environment or experimental handling procedures are vulnerable to oxidation. However, we show that the mass spectra intensity ratio of oxidized to non-oxidized methionine residues in serum tryptic proteins can be accurately quantified using a single drop of human serum and give stable and reproducible results. Our data demonstrate that two methionine residues in serum albumin (Met-111 and Met-147) are highly oxidized to methionine sulfoxide in patients with diabetes and renal failure and in healthy smokers versus non-smoker controls. This label-free mass spectrometry approach to quantify redox changes in methionine residues should facilitate the identification of additional circulating biomarkers suitable for predicting the development or progression of human diseases.

Oxidative stress is a common component of the pathophysiology of a variety of human conditions and diseases, including aging^1^,^2^, cancer^3^, diabetes^4^ and neurodegenerative^5^ and cardiovascular diseases^6^. Oxidative modifications of biomolecules, such as lipids, proteins and DNA, are postulated to contribute to the development and/or progression of the listed conditions. Determination of *in vivo* oxidative stress status requires quantification of either reactive oxygen/nitrogen species or damaged biomolecules. Because the former have very short half-lives and show high chemical reactivity, free radical processes have been monitored by detecting the latter. The identification of clinical biomarkers that accurately evaluate the severity of oxidative stress remains an important unmet challenge^7^.

Methionine (Met) oxidation is a mechanism by which proteins perceive oxidative stress and function in redox signaling^8^. Met residues are highly susceptible to modification by mild oxidants^8^ and can be oxidized spontaneously during common experimental procedures^9^. *In vitro* Met oxidation is a reversible process^10^,^11^ and is dependent upon solvent accessibility^12^,^13^ and structural determinants^14^,^15^. Met oxidation can also modify the physicochemical properties of the whole protein and therefore modulate its function^14^,^16^. However, the biological implications of the presence of oxidized Met in specific disease-related proteins have only been studied in limited numbers of human pathologies, including diabetes^17^, skin disease^10^,^11^, Alzheimer’s disease^18^ and Parkinson’s disease^19^. Mass spectrometry has emerged as a powerful tool to identify protein modifications in biological samples, but no adequate proteomic methodologies exist to characterize the exact sites and extent of oxidation in the peripheral circulation.

We have established a series of new technologies to enrich and analyze circulating low-molecular-weight peptides suitable for mass spectrometry analysis and have comprehensively identified native and tryptic peptides in the human peripheral circulation^20^,^21^,^22^,^23^. Among the large number of native peptides we recently identified, ~3% contained post-translationally oxidized Met residues. These results prompted us to develop a new label-free mass spectrometry approach that would enable identification of tryptic peptides containing Met residues that had undergone oxidative modification in serum. Such analysis of post-translational modification has been thought to be extremely challenging, but we demonstrate that this method is applicable to the assessment of whole-body oxidative stress status using a single drop of human serum.

## Results

### Selection of serum tryptic peptides containing an oxidized methionine

**Figure 1:**
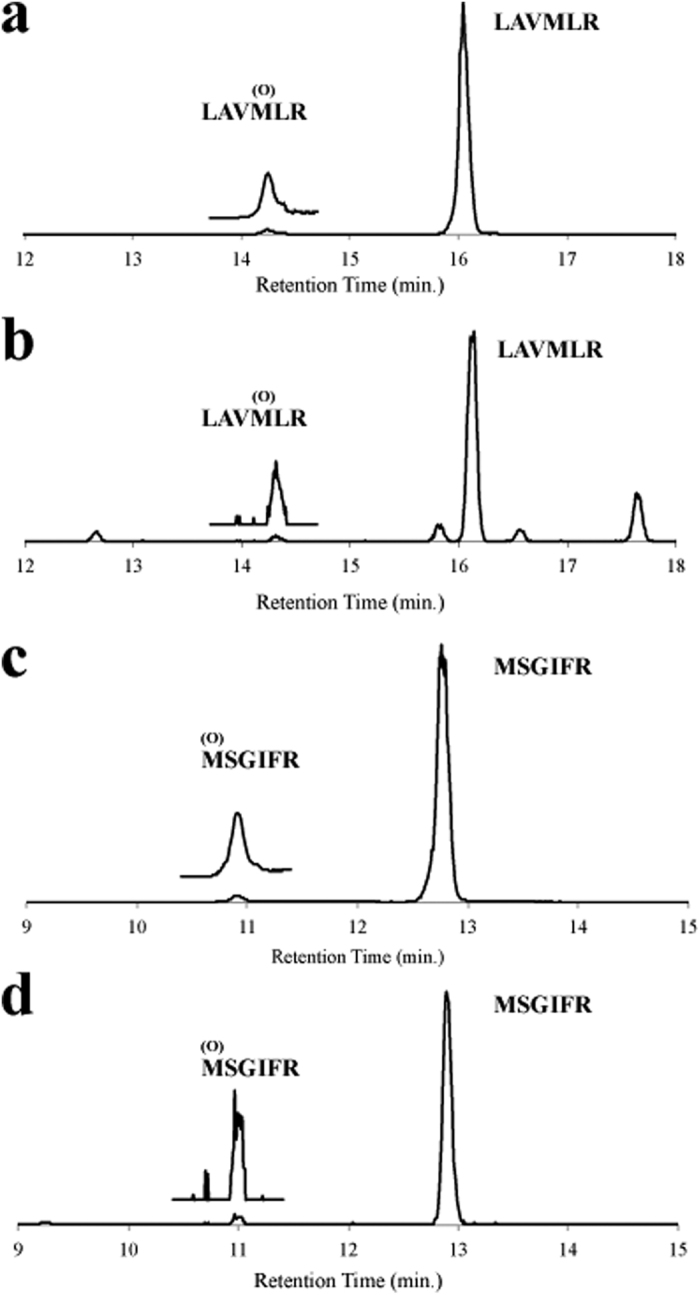
Comparison of methionine oxidation profiles before and after spiking peptide into human serum, reductive alkylation and trypsinization. Trypsin-digested *E. coli* β-galactosidase (2.5 μg/μl serum) was added to human serum as a monitor peptide and subsequently processed by reductive alkylation and trypsinization. Representative extracted ion chromatograms corresponding to the peptides LAVMLR (**a,b**) and MSGIFR (**c,d**) of the tryptic *E. coli* β-galactosidase peptide before (**a,c**) and after (**b,d**) spiking, reductive alkylation and trypsinization, are presented. Smaller peaks with an earlier retention time, corresponding to LAVMLR or MSGIFR peptides containing methionine sulfoxide (designated as (O) above M) were magnified 10-fold and are shown above the original peaks.

Because Met residues are reportedly vulnerable to oxidation in many experimental settings, we first examined whether unoxidized Met residues in tryptic peptides spiked into human serum can be oxidized into methionine sulfoxide (Met(O)) during the processes of reductive alkylation, trypsin digestion and subsequent analysis by liquid chromatography-mass spectrometry (LC-MS). We quantified signal intensities of peptides with Met(O) ([Met(O)]) and those with corresponding unoxidized Met ([Met]) using extracted ion chromatograms of LC-MS analysis and found that there were no significant differences in [Met(O)]/[Met] ratios when quantified before and after processing of human serum samples containing tryptic *E. coli* β-galactosidase peptides (Fig. 1).

**Figure 2:**
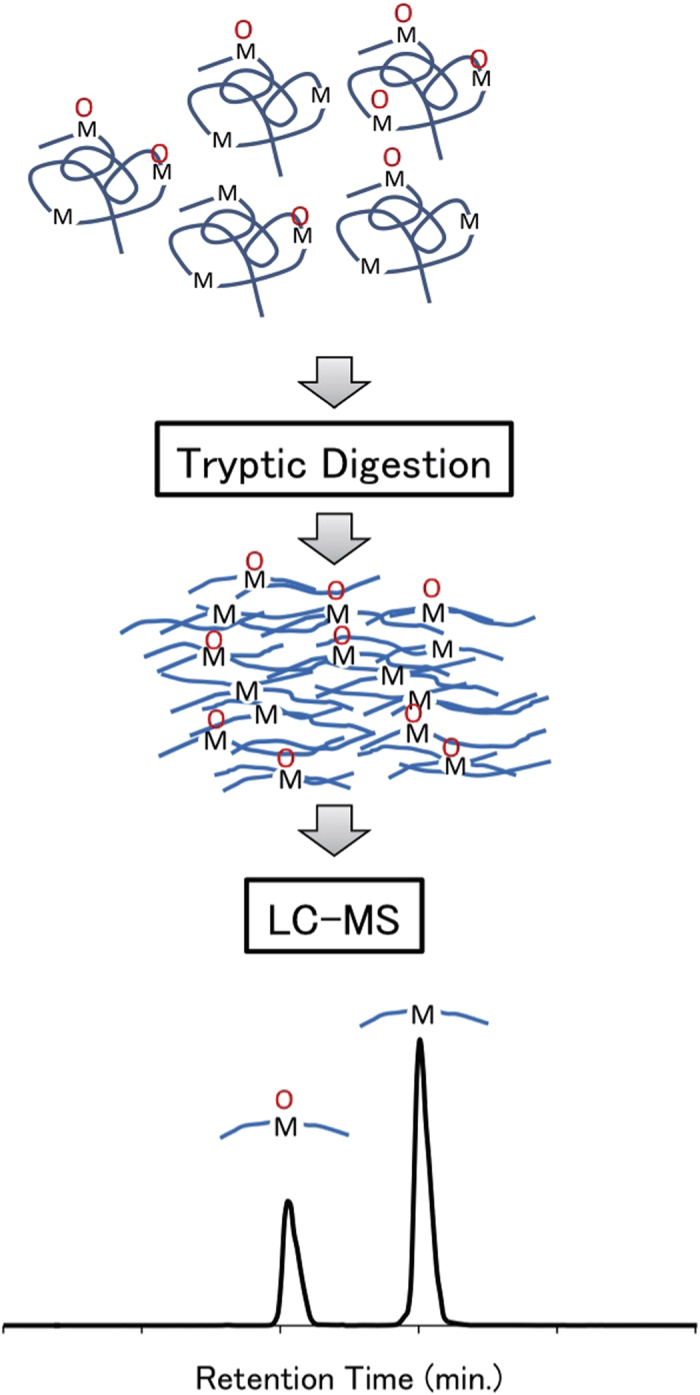
Schematic overview of the label-free mass spectrometry approach for quantifying oxidized and unoxidized methionine residues. After serum separation, proteins were digested to peptides without the use of depletion columns, and subjected to liquid chromatography-mass spectrometry (LC-MS) analysis to identify abundant tryptic peptides containing methionine residues. Tryptic peptides with unoxidized methionines and those containing methionine sulfoxide showed distinct retention times, as shown in the lowest panel. “M” within a blue curve denotes a methionine residue in a serum tryptic peptide and a red circle above the “M” represents the sulfoxide bond on a methionine residue.

We next determined whether [Met(O)]/[Met] for serum tryptic peptides is stable and can be quantified reproducibly using the study workflow summarized in Fig. 2. Initially, serum samples obtained from healthy controls were digested overnight with trypsin and subjected to highly sensitive analysis using nano-flow LC-MS. This resulted in identification of 53 peptides containing Met(O). Using LC-MS incorporating conventional HPLC, we selected potential biomarker candidates for further analysis based on the following criteria: that they were tryptic peptides containing Met and/or the corresponding Met(O) and possessing sufficient mass spectrum intensity, that the mass spectra of both peptides were clearly separable from other peptides, that mis-cleavage products were not detected and that determination of [Met(O)]/[Met] after repeated measurements of 12 aliquots showed inter-assay coefficients of variation (CVs) of less than 30%. Five Met-containing tryptic peptides fulfilled these criteria at this initial assessment (Table 1). Of these, more than 99.9% of serum complement C3 (Met-1118) was oxidized to Met(O) in all samples examined and therefore this peptide was excluded from further analysis. The remaining four tryptic peptides, albumin (Met-111), albumin (Met-147), immunoglobulin (Ig)γ1 chain C region (Met-135) and α1-antitrypsin (Met-409), were subjected to subsequent detailed analyses for potential application as clinical biomarkers.

### Optimization of a method for quantifying methionine oxidation

**Figure 3:**
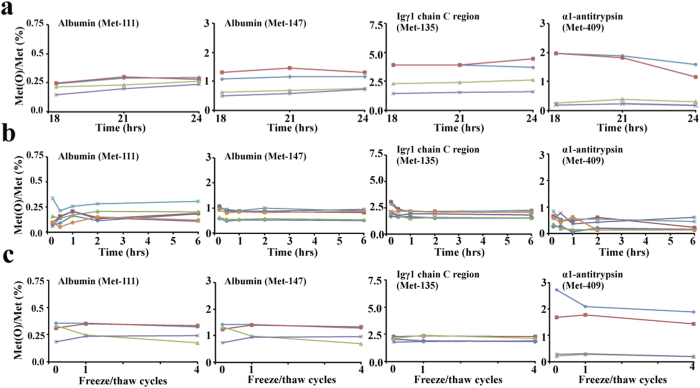
Effects of blood sample handling procedures on methionine oxidation in four serum tryptic peptides. **(a)** Effects of trypsin digestion time on ratio of mass spectrum signal intensity of peptide containing oxidized methionine residue to that of unoxidized methionine ([Met(O)]/[Met]) in the four candidate tryptic peptides. Blood samples withdrawn into serum separator tubes were left undisturbed at room temperature for at least 30 min, separated by centrifugation and, after reductive alkylation, digested with trypsin for 18–24 h. Samples were then subjected to LC-MS analysis to determine [Met(O)]/[Met] for the four tryptic peptides. **(b**) Effect of clotting time on [Met(O)]/[Met] in the four tryptic peptides. Blood samples were either immediately centrifuged after withdrawal or left undisturbed at room temperature for the indicated times, before centrifugation to separate serum. The sera obtained were reductively alkylated, digested with trypsin for 24 h and subjected to LC-MS analysis to measure [Met(O)]/[Met]. **(c)** Effects of freeze/thaw cycles on [Met(O)]/[Met] levels in the four tryptic peptides. Serum samples obtained by centrifugation of blood samples that had been left undisturbed for 30–60 min were trypsinized for 24 h and subjected to LC-MS analysis before and after repetition of the indicated numbers of freeze/thaw cycles. [Met(O)]/[Met] of the four tryptic peptides were determined. The numbers attached to each Met represent the amino acid position of the methionine residue in the UniProt protein database. Each line corresponds to one plasma donor.

To accurately quantify the frequency of Met(O) residues relative to their corresponding unoxidized residues, complete trypsin digestion is mandatory prior to LC-MS analysis. Trypsin treatment reduced the number of undigested products as a function of time, at least during the initial period (data not shown), but from 18 hours onwards, mis-cleavage products were undetectable and did not significantly affect [Met(O)]/[Met] in any of the four candidate serum tryptic peptides (Fig. 3a). Therefore, all serum samples were trypsin-digested for 24 hours in the experiments performed thereafter.

We next tested whether the rate of spontaneous oxidation of Met residues in serum proteins would be affected by the length of clotting time necessary to obtain serum, given that withdrawn blood samples are left for at least 30–60 min at room temperature for a clot to form and residual cellular elements and other contaminants to be removed. To investigate this, blood samples were either immediately centrifuged after blood withdrawal, or allowed to clot at room temperature for 30 min to 6 hours before centrifugation, and the sera obtained were processed for reductive alkylation, trypsin digestion and subsequent LC-MS analysis. We found that [Met(O)]/[Met] of the four tryptic peptides were very similar, irrespective of the time the blood samples were left at room temperature (Fig. 3b).

We also compared [Met(O)]/[Met] of the four peptides before and after freeze/thaw procedures. Repetition of freeze-thaw up to four times did not affect [Met(O)]/[Met] levels in any of the peptides (Fig. 3c). Thus, subsequent clinical analyses employed serum samples obtained by routine blood handling procedures that had been stored at −30 °C, as described in Materials and Methods.

### Oxidized methionine in serum proteins fluctuates minimally in association with physiological states but is altered in certain disease states

**Figure 4:**
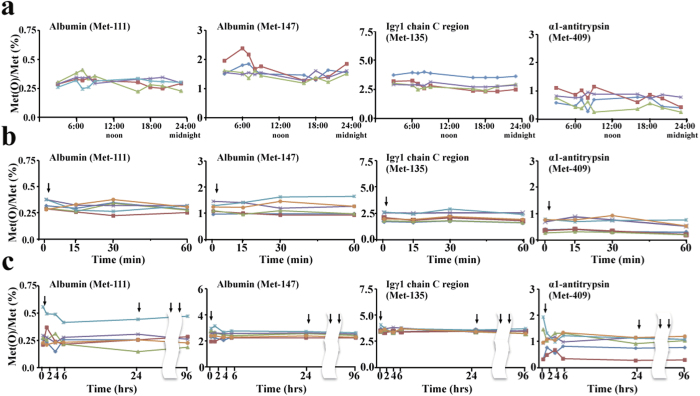
Diurnal variation and acute effects of smoking and oral vitamin C intake on methionine oxidation in serum proteins. **(a)** Circadian changes in [Met(O)]/[Met] levels of the four candidate tryptic peptides in healthy non-smokers. Serum samples were obtained from four healthy volunteers at the indicated time of day from a catheter placed into an antecubital vein, trypsinized and analyzed by LC-MS to determine [Met(O)]/[Met] for each peptide. **(b)** Effects of acute smoking on [Met(O)]/[Met] levels of the four tryptic peptides in healthy smokers. Serum samples were obtained from a catheter placed into an antecubital vein of six healthy smokers before and 15, 30 and 60 min after smoking two cigarettes, and after reductive alkylation and trypsinization, [Met(O)]/[Met] levels of the four tryptic peptides were determined. **(c)** Effects of oral vitamin C intake on [Met(O)]/[Met] levels of the four tryptic peptides. Serum samples were obtained from six healthy volunteers before and 2, 4, 6 and 24 h after ingesting a 1000-mg vitamin C tablet on the first morning. The subjects then ingested the same dose of vitamin C daily for the next 3 successive days and another serum sample was collected on the fifth morning. [Met(O)]/[Met] for the four tryptic peptides were determined in these serum samples. Numbers attached to each Met represent the amino acid position of the methionine residue in the UniProt protein database. Arrows indicate the times during which smoking and vitamin C ingestion took place. Each line corresponds to one plasma donor.

Baseline [Met(O)]/[Met] of the four candidate serum tryptic peptides in healthy non-smokers ranged from 0.252 ± 0.057% (albumin (Met-111)) to 2.511 ± 0.814% (Igγ1 chain C region (Met-135)), while that of complement C3 (Met-1118) was always higher than 99.9% in all subjects. Thus, distinct baseline oxidative statuses are apparent for each methionine residue. [Met(O)]/[Met] levels of the other four peptides determined nine times a day (3:00, 6:00, 7:00, 8:00, 9:00, 16:00, 18:00, 20:00 and 23:00) in four healthy volunteers showed minimal diurnal fluctuations (Fig. 4a).

We determined [Met(O)]/[Met] of these four peptides in six healthy smokers before and after cigarette smoking. Acute smoking did not affect [Met(O)]/[Met] of any of the four peptides (Fig. 4b). In addition, six non-smokers took 1000 mg vitamin C tablets for 4 successive days, but their [Met(O)]/[Met] levels did not show any significant changes as a result (Fig. 4c).

**Figure 5:**
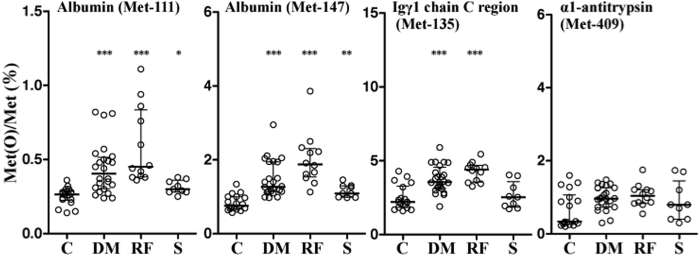
Whiskerplots showing serum levels of methionine sulfoxide relative to unoxidized methionine in the four candidate serum tryptic proteins in healthy non-smokers, patients with type 2 diabetes, patients with diabetes-associated renal failure and healthy smokers. Serum samples obtained from healthy volunteers and type 2 diabetic patients with or without renal failure were digested with trypsin for 24 h and subjected to LC-MS analysis to determine [Met(O)]/[Met] of the four tryptic peptides. Numbers attached to each Met residue represent its position according to the UniProt protein database. Horizontal bars indicate median and whiskers extending 25th and 75th percentiles of respective [Met(O)]/[Met] values. C denotes healthy non-smokers, DM denotes patients with type 2 diabetes without impaired renal function, RF denotes diabetic patients with non-dialyzed chronic renal failure and S denotes healthy smokers. One way ANOVA followed by Mann-Whitney U post hoc test was used to compare [Met(O)]/[Met] levels for each pair of groups. *P < 0.05; **P < 0.005 ***P < 0.0001.

We next measured [Met(O)]/[Met] levels in 23 patients with type 2 diabetes, 12 patients with diabetes-related chronic renal failure and nine healthy smokers, and compared these with levels in 18 healthy non-smokers (Table 2). Interestingly, [Met(O)]/[Met] for serum albumin (Met-111 and Met-147) and of Igγ1 chain C region (Met-135) were high in type 2 diabetic subjects with normal renal function and in those with renal failure, compared to non-smoking control subjects (Fig. 5). Healthy smokers also showed significantly higher [Met(O)]/[Met] values in serum albumin (Met-111 and Met-147) than healthy non-smokers (Fig. 5). [Met(O)]/[Met] levels in α1-antitrypsin (Met-409) were not different between any pairs within the four subject categories. Multivariate analyses confirmed that presence of diabetes and renal failure was independently associated with higher [Met(O)]/[Met] values of serum albumin (Met-111) (β = 0.368, F = 13.893, P < 0.001; β = 0.269, F = 7.180, P = 0.0095, respectively) and albumin (Met-147) (β = 0.589, F = 24.942, P < 0.001; β = 0.375, F = 13.551, P < 0.001, respectively) and that diabetes and smoking significantly impacted higher [Met(O)]/[Met] levels of Igγ1 chain C region (Met-135) (β = 0.601, F = 25.376, P < 0.001) and serum albumin (Met-147) (β = 0.281, F = 6.715, P = 0.012), respectively. Gender and age were not selected as independent variables influencing any of the four [Met(O)]/[Met] values in the multivariate analysis.

Because high glycemic variability induces more oxidative stress than continuous high glucose^24^, we analyzed the glycemic excursion profiles of our diabetic patients using continuous glucose monitoring (CGM) at the time of blood sampling and studied whether [Met(O)]/[Met] values are related to mean glucose or glycemic standard deviation (SD) data calculated using all 576 glucose values obtained at every 5-min intervals during 48-h CGM recordings^25^. Patients with glycemic SD values in the upper tertile (54–93 mg/dl, age 65.8 ± 10.8, n = 12) had higher [Met(O)]/[Met] of serum albumin (Met-111) than those in the lower SD tertile (20–42 mg/dl, age 72.2 ± 10.8, n = 11) (0.586 ± 0.275%, vs. 0.395 ± 0.098%, P < 0.05). HbA1c levels were not significantly different between the patients in the upper and lower glycemic SD tertile (HbA1c 8.9 ± 2.7%, vs. 7.7 ± 1.6%, ns). The upper tertile of diabetic patients categorized using CGM mean glucose, HbA1c or glycated albumin levels did not have higher [Met(O)]/[Met] in any four tryptic peptides than the corresponding lower tertile patients.

## Discussion

Met is readily oxidized to Met(O), and this reaction is believed to be reversibly catalyzed by the methionine sulfoxide reductases^26^ present in nearly all organisms^27^,^28^. Met(O) can also be further oxidized to methionine sulfone, although this reaction occurs to a much lesser extent^29^,^30^,^31^. Despite these well-known confounding factors regulating Met oxidation and the claimed technical difficulties associated with quantifying peptides containing Met and Met(O), the present study has indicated that [Met(O)]/[Met] of specific residues may be promising clinical biomarkers for oxidative stress.

Three lines of evidence support this proposal. First, [Met(O)]/[Met] in some serum tryptic proteins were accurately determined using mass spectrometry in a highly reproducible manner. Although baseline steady-state [Met(O)]/[Met] levels detected in the current study widely varied among particular methionine residues, the values were neither affected by clotting time at room temperature for serum separation, nor by serum handling procedures, such as reductive alkylation, trypsin digestion, repeated freeze-thaw, and subsequent mass spectrometry analysis. Second, [Met(O)]/[Met] in all four candidate serum tryptic proteins showed limited diurnal fluctuation and negligible acute changes after smoking or ingestion of vitamin C, a well-known physiological antioxidant. Third, two Met residues in serum albumin showed elevated [Met(O)]/[Met] in patients with type 2 diabetes, diabetes-associated chronic renal failure and smokers, reflecting the altered oxidative stress status present in these conditions. Thus, some Met residues in serum proteins may undergo oxidation far more easily than other residues in response to underlying diseases or conditions.

Experience in many areas of redox research highlights how challenging it can be to identify clinical biomarkers reflecting endogenous oxidative stress status. The experimental and analytical performance of [Met(O)]/[Met] measurement was reproducible and robust, irrespective of the time of blood withdrawal, the time the sample was left at room temperature before centrifugation or repeated freeze/thaw cycles. Such results may be attributable to a previously reported feature of the ubiquitously expressed enzymes regulating Met(O) levels. Met(O) reductase, for example, may not function in the presence of circulating proteins^19^,^32^, and hence the oxidation status of serum proteins with intravascularly oxidized Met residues remains unchanged until the proteins are broken down or cleared, predominantly by the liver in the case of serum albumin.

Our LC-MS approach has overcome well-recognized technical difficulties associated with quantifying redox changes in circulating proteins. Incomplete digestion of serum proteins affected [Met(O)]/[Met] due to differential digestion efficiency between unoxidized Met and Met(O), but we have achieved complete trypsin digest by using phase-transfer surfactant plus Lys-C^33^. Stable and reproducible LC-MS analyses were achieved over a long run time by employing conventional HPLC equipped with metal needle spray tips with a typical flow rate of 200 μL/min, rather than by using capillary HPLC equipped with glass capillary spray tips, which are commonly used to attain high sensitivity analysis of proteins and peptides. Hence, the technical and physiological variability in [Met(O)]/[Met] of serum albumin (Met-111 and Met-147) and of Igγ1 chain C region (Met-135) appears to be minimal compared with the pathophysiologically significant redox changes to their Met residues in the human peripheral circulation. Given its rapidity, simplicity and cheapness, wide applications of this label-free mass spectrometry approach may be possible to identify surrogate biomarkers that reflect intravascular redox status and predict future development and/or progression of a variety of human diseases.

Our data showed distinct variation in the baseline steady-state oxidation status between candidate Met residues. For example, Met-111 and Met-147 of serum albumin showed far lower baseline [Met(O)]/[Met] than Met-1181 of complement C3, but these residues were clearly more oxidized in patients with type 2 diabetes and renal failure. Met-111 of serum albumin showed distinctly higher [Met(O)]/[Met] in diabetic patients with greater glycemic excursion than those with minimal variability. These results indicate that these Met residues are susceptible to oxidation by factors that are associated with the pathophysiology of such diseases. Accumulating evidence indicates elevated oxidative stress in diabetes^34^,^35^,^36^, and further increased oxidative stress and risk for cardiovascular disease when nephropathy is also present^37^,^38^,^39^. Acute fluctuations of glucose levels in diabetes is demonstrated to accelerate oxidative stress^24^. Cigarette smoke contains high concentrations of free radicals that can induce oxidative stress^40^,^41^ and smokers have a higher prevalence of variety of diseases, including respiratory diseases, cardiovascular diseases and cancers^42^. Our results show that although healthy smokers possess higher [Met(O)]/[Met] with respect to Met-111 or Met-147 of serum albumin, the magnitudes of the increases in healthy smokers are less than those in diabetic and renal failure patients. Because acute smoking did not change [Met(O)]/[Met] in any of the four peptides tested, longer term oxidative stress may be required to oxidize Met residues of proteins in the human peripheral circulation.

In conclusion, we have developed a novel label-free mass spectrometry approach to quantify Met oxidation in proteins present in the human peripheral circulation. Oxidized Met levels were accurately and reproducibly determined in a single drop of human serum. [Met(O)]/[Met] levels of two residues in serum albumin were elevated in type 2 diabetes, diabetes-associated chronic renal insufficiency and chronic smoking. This strategy could also be applied to identify other Met-containing serum proteins whose status reflects the severity of pathophysiological conditions or the likelihood of a patient developing a major disease in the future.

## Methods

### Subjects

The study population consisted of 18 healthy non-smokers (9 men and 9 women), 23 patients with type 2 diabetes (14 men and 9 women), 12 patients with non-dialyzed chronic renal failure associated with type 2 diabetes (9 men and 3 women), and 9 healthy smokers (9 men) (Table 2). Type 2 diabetes was diagnosed in individuals who were confirmed to be insulin independent according to the criteria of the Japan Diabetes Society (patients with anti-glutamic acid decarboxylase autoantibody >1.5 U/ml or serum C-peptide <0.5 ng/ml were excluded)^43^. Chronic renal failure was diagnosed after confirming sustained elevation of serum creatinine levels for at least 6 months^44^,^45^. Clinical records were reviewed for all subjects and those with histories of acute inflammatory diseases, malignancies, recent attacks of cerebrovascular or cardiovascular accidents were excluded from the analysis. The protocol was approved by the Kitasato University Medical School Ethics Committee (B15–181) and informed written consent was obtained from all participants. All study methods were performed in accordance with the relevant guidelines and regulations of Kitasato University Medical School.

### Serum sample collection

Venous blood samples were collected from healthy volunteers and patients with type 2 diabetes with and without impaired renal function. Type 2 diabetic patients provided an additional serum sample at the time of scheduled blood sampling on the last morning of 72-h CGM recording using the CGMS system GOLD (Medtronic Minimed Inc. Northridge, CA)^46^,^47^. They also received our routine systemic evaluation protocols for diabetic patients covered by universal health coverage system in Japan^43^,^48^, which include laboratory tests, such as urinalysis, a complete blood count, serum biochemical analysis for more than 15 items, anti-glutamic acid decarboxylase antibody, glycated albumin, HbA1c, and fasting serum insulin. Blood was withdrawn from an antecubital vein into vacutainers containing pro-coagulant and allowed to clot at room temperature for the indicated time, before centrifugation at 2,000 × *g* for 15 min at room temperature. Aliquots were stored at −30 °C until use.

### Trypsin digestion of serum samples

Trypsin digestion of serum proteins was performed essentially as described^33^, with the following modification. Twenty μL of 200 mM triethylammonium bicarbonate/12 mM sodium deoxycholate/12 mM sodium lauryl sulfate and 2 μL 200 mM tris (2-carboxylethyl) phosphine hydrochloride/120 mM triethylammonium bicarbonate were added sequentially to thawed serum (2 μl) and this was incubated at 50 °C for 30 min. Two μl of 375 mM iodoacetamide was added, the mixture was incubated in a dark room for 30 min, then further incubated at 37 °C for the indicated times after addition of 2 μl of 100 ng/μl Lys-C and 2 μl of 100 ng/μl trypsin. 50 μl of acetonitrile (ACN) and 50 μl of 5% trifluoroacetic acid were then added to the digest, this was centrifuged at 19,000 g for 15 min, and the supernatant was subjected to LC-MS analysis.

### Identification of methionine-containing peptides by LC-MS analysis

Tryptic digests of serum samples were injected onto a C_18_ 0.075 × 20 mm trap column (Acclaim PepMap 100; GL Sciences, Tokyo, Japan) and then eluted onto a C_18_ 0.075 × 120 mm analytical column (Nano HPLC Capillary Column; Nikkyo Technos, Tokyo, Japan), configured to an EASY-nLC 1000 HPLC system (Thermo Fisher Scientific). The flow rate of the mobile phase was 300 nL/min; mobile phase A consisted of 0.1% formic acid (FA) and mobile phase B consisted of 0.1% FA/90% ACN. The mobile phase gradient was programmed as follows: 5–25% B (0–48 min), 25–50% B (48–58 min), 50–95% B (58–60 min), and 95% B (60–70 min). Separated peptides were introduced from the HPLC to a Q-Exactive (Thermo Fisher Scientific) operating in data-dependent mode, to automatically switch between full-scan MS and MS/MS acquisition. Full-scan MS spectra (*m/z* 350–1,000) were acquired in an Orbitrap instrument with a mass resolution of 70,000 at *m/z* 200, after accumulation of ions to a 1 × 10^5^ target value. Fractions representing the 12 most intense full-scan peaks with charge state 2–4 were selected using an isolation window of 2.4 Da and fragmented in the high-energy collisional dissociation cell at a normalized collision energy of 27%. MS/MS spectra were acquired by the Orbitrap mass analyzer with a mass resolution of 17,500 at *m/z* 200, after accumulation of ions to a 1 × 10^5^ target value. The ion selection threshold was 2 × 10^4^ counts, and the maximum allowable ion accumulation times were 120 ms for full MS scans and 200 ms for MS/MS spectra. Typical mass spectrometric conditions were as follows: spray voltage 2 kV, no sheath or auxiliary gas flow, capillary temperature heated to 250 °C and dynamic exclusion time 15 s.

Database searches were performed using the SEQUEST algorithm incorporated into Proteome Discoverer 1.4.0.288 software (Thermo Fisher Scientific). The search parameters were as follows: enzyme, trypsin; variable modification, oxidation of Met residue; variable modification, carbamidomethylation of Cys residue; peptide ion mass tolerance, 6 ppm; fragment ion mass tolerance, 0.02 Da; peptide charges, +2 to +8. The identified peptides were searched for in the decoy database and the false discovery rate was set as 0.01 using Percolator scoring with posterior error probability validation. Peptide quantitation was also performed using Proteome Discoverer 1.4.0.288.

### LC-MS analysis of the oxidation ratio of methionine-containing peptides

To quantify [Met(O)]/[Met], digested peptides were injected onto a 2.0 mm (inner diameter) × 50 mm CAPCELL PACK MGIII-H S3 column attached to a Nanospace SI-2 HPLC system (Shiseido Fine Chemicals, Tokyo, Japan). The column temperature was maintained at 45 °C. The flow rate of the mobile phase was 200 μL/min; mobile phase A consisted of 0.05% FA and mobile phase B consisted of 0.05% FA/90% ACN. The mobile phase gradient was programmed as follows: 0% B (0–3 min), 0–55.5% B (3–40 min), 55.5–80% B (40–40.1 min), and 80% B (40.1–45 min). Peptides were introduced from the HPLC to an LTQ-Orbitrap Discoverer. Full-scan MS spectra (*m/z* 300–2,000) were acquired using an Orbitrap instrument at a mass resolution of 30,000 at *m/z* 400.

### Statistical analysis

Data are expressed as mean ± SD unless stated otherwise. Differences among groups or time-course changes in [Met(O)]/[Met] levels were analyzed using ANOVA. *Post hoc* comparisons were performed using Mann–Whitney U and/or Wilcoxon’s tests. Multivariate analyses were performed employing age, gender, diabetes, renal failure and smoking as an explanatory variable and each [Met(O)]/[Met] value as an objective variable. All analyses were performed with JMP ver. 5.0.1a (SAS, Cary, NC, USA). P < 0.05 was considered statistically significant.

## Additional Information

**How to cite this article**: Suzuki, S. *et al*. Methionine sulfoxides in serum proteins as potential clinical biomarkers of oxidative stress. *Sci. Rep.* **6**, 38299; doi: 10.1038/srep38299 (2016).

**Publisher's note:** Springer Nature remains neutral with regard to jurisdictional claims in published maps and institutional affiliations.

## Figures and Tables

**Table 1:** Five tryptic peptides containing a single methionine sulfoxide residue.

Protein Name	Sequence	Gene	UniProt	Sequence			*m/z*		charge
		Name	accession	start	end	Met	Met	Met(O)	
Serum albumin	ETYGE**M**ADCCAK	ALB	P02768	106	117	111	717.7703	725.7678	2
Serum albumin	LVRPEVDV**M**CTAFHDNEETFLK	ALB	P02768	139	160	147	884.0928	889.4245	3
Complement C3	AGDFLEANY**M**NLQR	C3	P01023	1172	1185	1181	821.3881	829.3856	2
Igγ1chain C region	DTL**M**ISR^*^	IGHG1	P01857	132	138	135	418.2207	426.2182	2
α1-antitrypsin	SPLF**M**GK	SERPINA1	P01009	405	411	409	390.2097	398.2071	2

*The same tryptic peptide could derive from the following four isoforms: IGHG1 (Met-135), IGHG2 (Met-131), IGHG3 (Met-182), and IGHG4 (Met-132).

**Table 2:** Clinical characteristics of the study population.

	Healthy non-smokers	Patients with type 2 diabetes	Patients with renal failure	Healthy smokers
Number (male/female)	18 (9/9)	12 (9/3)	23 (14/9)	9 (9/0)
Age (years)	49.9 ± 12.1	65.3 ± 11.2	68.4 ± 13.8	38.2 ± 5.4
HbA1c (%)	5.4 ± 0.2	8.4 ± 1.9	7.3 ± 2.5	5.5 ± 0.2
Serum creatinine (mg/dl)	0.75 ± 0.12	0.93 ± 0.26	5.82 ± 2.76	0.82 ± 0.08
